# A Tactical Medicine After-action Report of the San Bernardino Terrorist Incident

**DOI:** 10.5811/westjem.2017.10.31374

**Published:** 2018-02-26

**Authors:** Joshua P. Bobko, Mrinal Sinha, David Chen, Stephen Patterson, Todd Baldridge, Michael Eby, William Harris, Ryan Starling, Ofer Lichtman

**Affiliations:** * Loma Linda University, Department of Emergency Medicine, Loma Linda, California; † West County Special Weapons and Tactical Team, Orange County, California; ‡ First Care Provider Foundation, Research Associate; § Huntington Beach Special Weapons and Tactical Team, Huntington Beach, California; ¶ San Bernardino Sheriff Aviation Department, Division of Aviation, San Bernardino, California; || Adjunct Professor, Citrus College; # San Bernardino Police Department, San Bernardino, California; ** First Care Provider Foundation, Director; †† Rancho Cucamonga Fire District, Rancho Cucamonga, California

## INTRODUCTION

On December 2, 2015, terrorism landed in Southern California when two perpetrators aligned with the Islamic State (IS), murdered and wounded 38 civilians at the Inland Regional Center of San Bernardino, California. Military tactics from the battlefields of Iraq and Afghanistan became strategies employed by terrorist organizations against civilians and domestic law enforcement agencies,^1^ requiring first-responder agencies to adapt rapidly to threats that are often discussed but rarely encountered. We describe systemic lessons that should be applied by medical directors of law enforcement, and fire and emergency medical services (EMS) agencies during a large-scale tactical medicine response.

### Timeline

**10:58 A.M** - The first 911 call is received, reporting gunshots in the vicinity of the 1300 block of South Waterman Avenue.

**10:59 A.M** - Call is upgraded to “shots fired” at the Inland Regional Center (IRC) with a description of three suspects dressed in black and carrying assault rifles.

**11:00** - Two patrol units from the San Bernardino Police Department (SBPD) are dispatched to the scene.

**11:04 A.M** - First responding police units are on scene and make entry. At this point the two shooters have departed the building after wounding 36 people, of whom 14 would later die. The initial police teams encounter three deceased victims just outside of the building. Law enforcement quickly clears the ground floor of obvious threats before entering the conference room. Within minutes, local fire and EMS units arrive and stage nearby, and the San Bernardino County Sheriff’s Department rescue helicopter begins flying around the IRC.

**11:09 A.M** - The San Bernardino Police Special Weapons and Tactics (SWAT) Team, which was coincidentally conducting training locally, arrives with a 13-person team, including a SWAT medic. This is the first medical asset to reach the victims. The team medic, after supporting the SBPD SWAT team, performs an initial clearing of the IRC; and begins triaging more than 30 wounded civilians.^2^

**11:10 A.M** - The Sheriff Department’s rescue helicopter lands nearby and offloads two aviation medics. These medics were not wearing their Kevlar® personal protective equipment (PPE). The onboard crew chief takes position as airborne sniper cover.

**11:15 A.M** - A triage, mass-casualty staging location is established by the tactical commander, and the first floor is reported by SWAT to be secured. This notification triggers the evacuation of the wounded to the triage area. Two deceased victims are transported by law enforcement to a nearby medical center.

**11:17 A.M** - Trauma centers are notified of the event through ReddiNet, the emergency medical notification system used by all hospitals within the region. Prior to the official notification, a mobile intensive care nurse at Loma Linda had been informed unofficially via a cell phone call from a firefighter dispatched to the scene. Five patients were transported to Loma Linda University Medical Center (LLUMC) and six to Arrowhead Regional Medical Center (ARMC).^3^

The total time elapsed during the shooting itself is less than five minutes. Unknown to the responders at the time, the terrorists had planted an improvised explosive device (IED) – a backpack containing pipe bombs with a crude remote detonator – in the conference room. The IED did not explode. From the authors’ first-hand estimates, there were at least 30 additional rescue personnel in the IRC conference room at the same time the IED was present, and prior to the device being made safe.

In all, seven surrounding agencies and four SWAT teams converged on the IRC. All of the critical shooting victims were quickly dispatched to the closest regional facilities: LLUMC, an American College of Surgeons (ACS) Level I trauma center; and ARMC, an ACS Level II trauma center. All of the critically wounded arrived for definitive trauma care within 57 minutes of being wounded. Resident physician conferences were being held at both centers that day, allowing both facilities to rapidly mobilize trauma and critical care resources for potential incoming casualties.

**14:00 P.M.** - The medical response is further complicated by a bomb threat that was called into LLUMC. While this call turned out to be a hoax, it required a substantial diversion of resources to clear during a day when law enforcement and EMS personnel were already being pushed to their limits.

**15:07–15:13 P.M.** - Law enforcement personnel conduct a felony car stop, and suspects engage in shootout, which ultimately ends with the death of both suspects.

## LESSONS LEARNED

### Lesson 1: There is a distinct difference between a qualified SWAT paramedic and a paramedic responding as part of a rescue task force (RTF)

Paramedics embedded with SWAT teams are trained to coordinate with team movements within the hot (unsafe) zone, providing medical support for the team as it progresses. Conversely, the current paradigm is that EMS personnel can be trained to enter the warm (safe) zone to conduct rescue operations when escorted by law enforcement. However, paramedics familiar with the RTF model are neither equipped nor trained sufficiently to provide care while under a direct threat.^4^ While these skill sets overlap they are not synonymous, and medical directors must not assume tactical paramedics integrated with the law enforcement SWAT will provide a sufficient medical resource for an RTF model.

**Figure 1:**
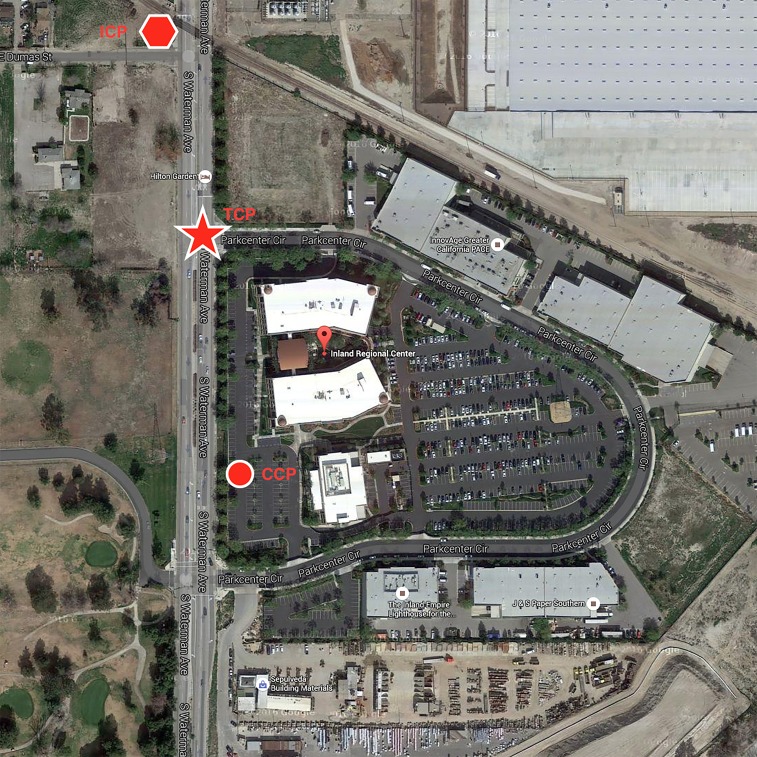
3D view of IRC with tactical positions. *ICP*, Incident Command Post; *TCP*, Tactical Command Post; *CCP*, Casualty Collection Point.

**Figure 2:**
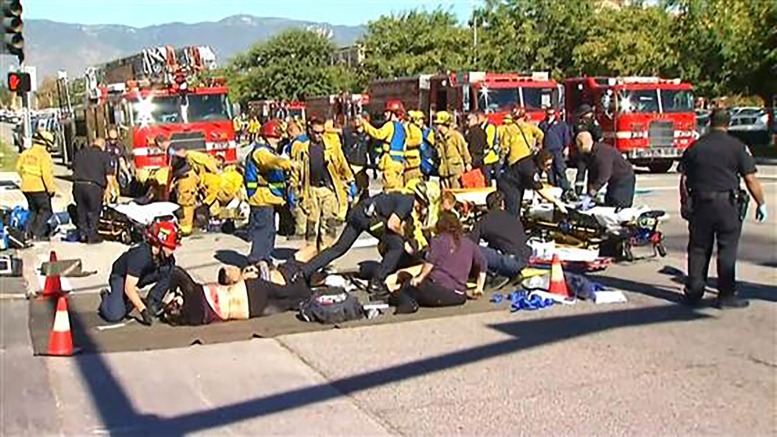
Casualty collection point.

The contrast between SWAT paramedics and RTF paramedics was highlighted in two ways. First, as the event unfolded, it became evident that responding fire and EMS units were not accustomed to combined operations with law enforcement. Their corresponding equipment packages and communications networks were different from those of the law enforcement responders. Furthermore, while clearly identified as an “active shooter” event by the first patrol units, the initial setup closely followed that of a mass casualty incident (MCI). The tactical command post was established to the north and the casualty collection area/treatment to the south (Figures 1 and 2). It is estimated that the south location was possibly within the blast radius of the IEDs left in the building. If this estimation was correct, by definition it means that the triage area was established in the hot zone (unsafe zone) and not on the warm/cold (safe zone) border as is traditionally taught. Regardless, in the presence of a dynamic threat it may become necessary to ensure traffic control to and create a perimeter for the treatment area (Figures 3 and 4).

Secondly, SWAT medics do not carry complete Advanced Life Support equipment due to their operational mandate for mobility. While they are often paramedics or physicians, their role as a SWAT medic is to provide medical aid only when operationally appropriate because their primary mission is to ensure the effectiveness of the law enforcement team. [The caveat is that a member of the public will receive priority because the duty of law enforcement is to ensure the safety and wellbeing of citizens.] Although a SWAT medic may enter deep within the hot zone with their tactical element, he or she does not carry equipment sufficient to provide sustained care for a large number of casualties in that zone. The support for ongoing evacuation care must come from follow-up resources, such as those provided by the RTF medical elements.^5^

Finally, within the current milieu of civilian, public, mass-shooting incidents, the latest data on civilian wounding patterns do not fit the prototype of the exsanguinating extremity injury, and thus are not amenable to the hemorrhage control techniques mastered by the tactical medic such as the use of tourniquets. These patients require rapid extrication, advanced resuscitation, and transport by a dedicated RTF component; they cannot be attended to solely by tactical medics.^6^

#### Learning Point #1

Recently, RTF has become a “buzz word” that first-responder departments use to demonstrate their effectiveness in tactical events. However, the role and implementation of such teams varies markedly from agency to agency. In practice, interoperability must continue to be emphasized by both command and ground-level units, and it must be practiced on a recurring basis to prevent confusion of operational objectives. On the day of the San Bernardino shooting only three fire agencies in the county had active RTF programs in place. Communication between these units was extremely strained by existing systems and the varied understanding of RTF concepts. Ensuring cohesive and coherent medical education across agencies will not only provide law enforcement with understanding of medical priorities, but also familiarize EMS with the tactical priorities of their law enforcement partners.

### Lesson 2: When possible, there should be a law enforcement medical coordinator (LEMC) within the command post structure

As many law enforcement agencies begin to deploy their own medical assets, it is critical that EMS medical directors recognize the tactical medical resource as separate from but augmenting the overall medical profile. This position falls outside the realm of the medical branch of the incident command system (ICS) because of its integration with operational teams. Thus, a law enforcement medical coordinator (LEMC) may provide a conduit to both EMS and fire assets as well as providing operational input to the incident commander.

The LEMC would then provide the commander with critical information that may be overlooked by the traditional medical branch of the ICS. First, the ability to conduct an in-depth, medical-threat assessment using operational data gathered by law enforcement and combined with EMS resources will provide on-scene commanders with a much better perspective on potential threats and limitations to operational plans.

Secondly, this position will provide improved integration between the tactical elements of the response and the force protection and rescue elements of the task force. Creating a LEMC position ensures proper allocation of both human and medical assets. Because SWAT medics operate within the law enforcement branch and not the medical branch, there is potential for duplication of efforts and general disorganization. This occurred in San Bernardino. Despite the traffic management by the SBPD, local resources pouring into the area of the shooting caused an obstacle to staged EMS assets. Medical resources were also being dispatched in duplicate with their respective law enforcement teams. Consolidated coordination of these assets would improve law enforcement support as well as integration for agencies less experienced with the RTF model.

Ideally this position would be filled by an active or former tactical medical provider – preferably a physician with knowledge of both the tactical and EMS functions. The benefits include continuous evaluation of the medical threat from law enforcement assets in the hot zone as well as EMS and fire in the warm/cold zone. Additionally, the LEMC would oversee resource need and distribution among the operational teams. Designating one individual streamlines the process and enables the SWAT medic to focus solely on providing emergent aid within the hot zone, while knowing that coordination is being managed by a professional who understands the scene, its evolution, and their needs.

Further, because of the uncertain nature of these operations, agencies must be prepared for extended operations.^7^ This possibility was understood by several teams present at the IRC event because they had recently been involved in the manhunt for Christopher Dorner, the disgraced Los Angeles Police Department officer who went on a shooting spree throughout Southern California. As the duration of that event extended several hours teams began to lack the basic necessities such as food and water, and experienced a shortage of personnel needed for the rotation system in order to sustain a high operational tempo. Though the logistics branch of the ICS is theoretically tasked with procurement of supplies for an operation, law enforcement team health remains under the purview of the tactical medic. Therefore, a LEMC would be the ideal person to ensure proper allotment of resources regardless of the duration of operations.

#### Learning Point #2

Because of the decentralized nature of SWAT resources during dynamic operations, a LEMC would assist the ICS with reducing or eliminating a conflicting medical response. This position would ideally be filled by an active or former tactical medical provider – preferably a physician with knowledge of both the tactical and EMS functions. The benefits include continuous evaluation of the medical threat to law enforcement assets in the hot zone, as well as EMS and fire in the warm/cold zone. Additionally, the LEMC would oversee resource need and distribution among the operational teams. Designating one individual streamlines the process and also enables the SWAT medic to focus solely on providing emergent aid within the hot zone while knowing that the coordination piece is being managed by a professional who understands the scene, its evolution, and their needs.

### Lesson 3: Modern terrorist events use a combination of multiple attackers, improvised weapons (e.g., IEDs), and occasionally centralized command and control

Law enforcement and fire departments have adapted quickly to minimize the loss of life in high-threat incidents through improved integration and education. Training for these scenarios is more often practiced as isolated events and less frequently combined. As a result, medical directors often outfit their teams in relation to the perceived threat, with PPE and medical equipment designed to protect from handguns and treat the “preventable causes of death.”

Despite this traditional mindset, it has been repeatedly demonstrated that modern terrorists coordinate complex attacks, using multiple detonations to “drive” response and inflict maximal damage. Although many of the victims of the San Bernardino terrorist event were shot numerous times, it has been well documented that there were unexploded IEDs in the immediate vicinity of both survivors and rescuers. In the face of multiple, armed attackers using high-powered rifles and multiple explosive devices, the typically-issued PPE is inadequate and the available medical supplies could quickly be exhausted, particularly when treating individuals with blast injuries.

Further, as active-shooter incidents have evolved, the push to incorporate Tactical Emergency Casualty Care (TECC) guidelines by first-responder agencies has accordingly focused on ballistic injuries. This approach emphasizes the need for hemorrhage control but overlooks both the likelihood of encountering victims with multiple amputations and the complications of blast injury not seen by a penetrating injury (which is only one of the components in a blast injury).

#### Learning point #3

Medical directors and medical assets should update their education programs to re-emphasize treatment of blast vs. ballistic injury. In addition, focused, mass-casualty management will help agencies and designated LEMCs as to the care and coordination necessary for adequate resource planning.^8^

In light of the threats now faced by our society, merely supplying one tourniquet, one chest seal and one dressing may no longer be sufficient. We recommend that ALL responders (including support personnel) carry tourniquets, while SWAT team members should carry several. In addition, designated law enforcement medical elements should wear the same PPE as their colleagues on patrol.

The development of a portable medical kit for active shooter/suspected terrorist events should be encouraged. Should extra equipment become necessary, this kit should contain multiple tourniquets, triage tape, combination dressing/bandages and large quantities of gauze for hemostasis/wound packing. Contrary to conventional thinking, establishment of an airway is not of primary concern in these types of events, eliminating the need for multiple advanced airway kits.

Most public buildings follow standard security practices, and medical directors and tactical medics should accordingly make basic changes in their response profiles. When the sprinklers were activated in the IRC building, medical assets were unprepared for operations in a wet environment. Moving forward, medical directors should educate and plan for the electrical shock hazards and biological hazards posed to responders in that environment. Rescue equipment should include waterproof triage tags (colored vinyl/plastic tags rather than paper), and teams should have the tools to circumvent difficulties with building access (traditionally law enforcement agencies have not had Knox Box® rapid entry system access) as part of the rescue plan. In the current environment, all tactical teams must have such access.

Finally, agency training can no longer accept notional acknowledgment to the presence of IEDs. The actual procedures for IED, complicated, active shooter incident (ASI) events should now be the standard, practiced scenario.^9^ Additionally, the complex and critical nature of injuries seen in these events and the challenge of accessing patients wounded by explosions, demonstrate the necessity for bystander care at the scene of the incident. Municipal and county agencies should consider training communities in TECC First Care Provider guidelines.^10^ Similarly, as the community has accepted the placement of automated external defibrillators (AEDs) in high-traffic areas, trauma/MCI equipment stations should also be pre-positioned in such areas and co-located with the AED.^11^

### Lesson 4: Despite several responders having military experience, there is a difference when witnessing catastrophic mortality within your own community

The brutal nature of the San Bernardino attacks and first responders’ familiarity with the community and the likelihood of recognizing victims are all powerful stimuli for the development of post-traumatic stress (PTS). Personnel who witnessed casualties within the main conference room were at significantly higher risk than those serving in other locations. In addition to mandatory critical incident stress (CIS) counseling, team medics immediately began interacting with team members to informally evaluate for signs of PTS.

#### Learning point #4

Stresses from these critical incidents may be reversed or halted through adaptive responses. Recognition that PTS is a likely outcome to mass casualty events should stimulate medical directors and team medics to create mechanisms for early recognition and practice of adaptive responses both for the individual and the collective. While individual stress is the focus of therapy, shared trauma or group stress remains a possible outcome. This shared trauma may unconsciously change processes within the group, affecting operational capabilities.^12^ Restricting access by non-essential personnel to victims remains the most basic process for decreasing stress in all groups.

Additionally, there is a marked difference to the responses expected by responding patrol units and organized SWAT units. While specialized teams may have the infrastructure to address PTS, including their own medical assets, individuals involved in the initial response may find it difficult to participate in departmental programs because they fear stigmatization. Avoidance of formal services may isolate and cause development of maladaptive responses that incur significantly higher risk for long-term pathology.^13^

Formal gatherings of team members and peer groups should be initiated very early to begin discussion of what has been witnessed and to prevent isolation by those most affected. However, support services must remain flexible and available to individuals reaching out to medical directors and team medics. Moreover, these gatherings must be protected from rules of discovery; fostering unguarded discussion/conversation is crucial to this process, and fear of retribution may destroy this process.

Finally, team medics may themselves need assistance following a crisis. It is imperative that medical directors or medical coordinators, as well as team leaders, allow for small-group or peer discussions in the aftermath of a critical event.

## CONCLUSION

Militarized terrorist tactics designed to inflict maximum damage to armored military units are now being employed by terrorists and purveyors of violence against unprotected civilian targets and domestic law enforcement agencies. These tactics, directed at civilians, are causing first responders to adapt at a rapid rate. For example, the classical law enforcement tactic of establishing perimeters and initiating negotiation, which was once considered optimal, is now lower priority compared to stopping active shooters as quickly as possible.

The experiences during and since the San Bernardino attacks of December 2, 2015, have changed the tactics, techniques and procedures of all law enforcement teams involved in the event. This evolving threat challenges first responders in California and across the world to revise and modify medical response to catastrophic events in novel and innovative ways.

Complex, coordinated attacks appear to be the new norm, and they require rapid adaptation in response tactics. Although the breadth and depth of lessons learned from the San Bernardino attacks are beyond the scope of a single paper, the lessons highlighted here provided a stimulus for discussion among the various stakeholders – EMS, law enforcement, EMS medical directors, and the public – about appropriate response in these types of events.

## Figures and Tables

**Table:** A visual correlation of lessons learned with problems encountered on December 2, 2015.

Problem/obstacle	Lesson learned
Recognizing the differences in capabilities between a SWAT medic vs. RTF medic/EMS	SWAT medics may be forward deployed; however, they will have limited resources and focus vs. EMS, which is able to provide more resources but will be unable to safely work closest to the point of injury or near an active/direct threat.
Coordination and deconfliction of EMS resources with law enforcement response in a large event	Establishing a law enforcement medical coordinator at the command post may provide a conduit to both EMS and fire assets as well as providing operational input to the incident commander.
Early forward deployment of amassed EMS resources for life saving measures	Regular RTF training that is cohesive and coherent across agencies will not only provide law enforcement with understanding of medical priorities, but also familiarize EMS with the tactical priorities of their law enforcement partners.
Extended duration of operations	Early planning for personnel rotation/substitution and for providing basic necessities such as food and water to sustain a high operational tempo and sustainment during a prolonged event.
Addressing the future of complex/coordinated attacks	Integrated, scenario-based training for LE and EMS. Recognizing the increasing IED threat and having the resources and training to treat multiple patients with blast injuries and multiple amputations.
Depletion of medical supplies in a multiple casualty event	Forward deployment of medical “4th man bag” stocked with TQs, chest seals, dressings, triage cards/tape in significant quantities. Operators and LE should carry multiple TQs in addition to their IFAKs.
Activation of sprinklers and klaxons and access within a structure	Educate and plan for the electrical shock hazards and biological hazards posed to responders. Waterproof triage tags/colored tape, Knox Box® access for rescuers.
Delay in treating victims with potentially survivable injuries	Training for members of the community to initiate bystander care (TECC First Care Provider guidelines) prior to arrival of EMS. Placement of trauma/MCI equipment stations co-located with AED’s in public spaces.
Addressing post-traumatic stress for rescuers, first responders, survivors and witnesses	Recognizing the need for and providing critical incident stress counseling. Team medics at the first opportunity should interact with team members to informally evaluate for signs of post traumatic stress. Providing formal and informal grief/crisis counseling post event.

*LE*, law enforcement; *SWAT*, special weapons and tactical team; *RTF*, rescue task force; *EMS*, emergency medical services; *IED*, improvised explosive device; *TQ*, tourniquet; *IFAK*, individual first-aid kit; *TECC*, tactical emergency casualty care; *MCI*, mass casualty incident; *AED*, automated external defibrillator
